# Decreased Balance Function in School-Aged Children with Behavioral Problems

**DOI:** 10.3390/brainsci12010117

**Published:** 2022-01-16

**Authors:** Naomichi Matsunaga, Tadashi Ito, Yuji Ito, Jun Mizusawa, Yingzhi Gu, Shota Sanada, Yuya Shirai, Daiki Takahashi, Nobuhiko Ochi, Koji Noritake, Hideshi Sugiura

**Affiliations:** 1 Department of Integrated Health Sciences, Graduate School of Medicine, Nagoya University, Nagoya 461-8673, Japan; matsunaga.naomichi@icloud.com (N.M.); sanjigen@mikawa-aoitori.jp (T.I.); gu.yingzhi@a.mbox.nagoya-u.ac.jp (Y.G.); sanada-shota@toyohashi-mh.jp (S.S.); shirai.yuya@k.mbox.nagoya-u.ac.jp (Y.S.); takahashi.daiki@g.mbox.nagoya-u.ac.jp (D.T.); 2 Three-Dimensional Motion Analysis Room, Aichi Prefectural Mikawa Aoitori Medical and Rehabilitation Center for Developmental Disabilities, Okazaki 444-0002, Japan; 3 Department of Pediatrics, Aichi Prefectural Mikawa Aoitori Medical and Rehabilitation Center for Developmental Disabilities, Okazaki 444-0002, Japan; yuji.ito@med.nagoya-u.ac.jp (Y.I.); aoi2pochi@yahoo.co.jp (N.O.); 4 Department of Rehabilitation, Aichi Prefectural Mikawa Aoitori Medical and Rehabilitation Center for Developmental Disabilities, Okazaki 444-0002, Japan; mizusawa.jun@outlook.com; 5 Department of Orthopedic Surgery, Aichi Prefectural Mikawa Aoitori Medical and Rehabilitation Center for Developmental Disabilities, Okazaki 444-0002, Japan; noritake@mikawa-aoitori.jp

**Keywords:** balance function, behavioral problems, children, one-leg standing test, Strengths and Difficulties Questionnaire

## Abstract

Children with behavioral problems have a high risk of impaired motor performance. However, the characteristics of balance functions and their associations with behavioral traits are unclear in this population. This study aimed to evaluate balance functions and their relationships with the degree of behavioral problems in school-aged children. A total of 209 children, aged 6–10 years, were divided into two groups, those with and those without behavioral problems, using the Strengths and Difficulties Questionnaire (SDQ). Physical assessments included the one-leg standing test (OLST), the two-step test, and the five-times-sit-to-stand test. We compared the data between groups and assessed for correlations in terms of total difficulties and the SDQ subscale scores. Children with behavioral problems showed significantly reduced the OLST results (*p* < 0.001) and the two-step test results (*p* = 0.008). The five-times-sit-to-stand test results did not show significant differences between groups. The OLST results were significantly correlated with emotional symptoms (r = −0.22, *p* < 0.001), hyperactivity/inattention (r = −0.29, *p* < 0.001), peer relationship problems (r = −0.22, *p* < 0.001), and total difficulties (r = −0.32, *p* < 0.001). Meanwhile, the two-step test results showed no significant correlation with the SDQ scores. Children with behavioral problems have poor balance function, thereby increasing the risk for instability. This suggests that the balance function of children with behavioral problems needs to be considered.

## 1. Introduction

Behavioral problems are one of the most remarkable health concerns during childhood, affecting 5–10% of children with a male to female ratio of 2:1 in prepubertal children [1]. Behavioral problems in children, characterized as either internalizing or externalizing problems, may eventually lead to a reduced quality of life and secondary social disabilities [2,3]. These problems seem to persist into adolescence, and they could potentially become more serious with age [1].

The Strengths and Difficulties Questionnaire (SDQ) is a widely used screening tool for assessing the negative and positive behavioral attributes of children and adolescents aged 4–17 years [4]. The psychometric properties and user acceptability of the SDQ [5,6,7] are well established. Although the Child Behavior Checklist and other questionnaires are also useful for assessing behavioral problems, the SDQ has the advantage of fast and efficient access, thereby reducing the burden on the family members who answer the questionnaire [8].

There have been some reports suggesting a relationship between motor performance and behavioral problems assessed using the SDQ among children and adolescents. Among school-aged children, motor performance was associated with behavioral problems, likely due to various psychosocial mechanisms [9]. Among adolescents, behavioral problems correlated not with body composition and muscle strength, but rather with cardiorespiratory fitness [10].

Static and dynamic balance functions are important in performing daily activities and are related to the nervous system, including somatosensory perception. However, the relationship between these balance functions and behavioral problems has not been fully investigated in this population. It is particularly important to focus on school-aged children, as early assessment and intervention before adolescence may yield positive outcomes.

The purpose of this study was to evaluate balance functions and their relationships with behavioral problems among school-aged children using the SDQ. We hypothesize that decreased balance functions are correlated with the degree of behavioral problems and that these may be related to specific psychosocial factors in this population.

## 2. Materials and Methods

### 2.1. Participants

A total of 295 elementary school students, aged 6–10 years, from Okazaki City, Aichi Prefecture, Japan, who underwent medical check-ups from April 2018 to March 2020 were included in the study. Participants were recruited via the distribution of flyers. The medical checkup consisted of medical examinations, physical function tests, verbal intelligence tests, and questionnaires, including the SDQ. This was conducted by a multidisciplinary team consisting of one pediatric orthopedic surgeon, one pediatric neurologist, one physical therapist, and many research assistants. Physical function tests included both balance function tests and functional muscle strength tests. The latter were performed to rule out lower limb weakness as the cause of the imbalance.

The exclusion criteria were as follows: (1) an inability to complete all assessments and (2) the presence of orthopedic, neurologic, ophthalmologic, auditory, respiratory, or cardiovascular abnormalities that may have affected the results of the physical function tests. With the exclusion of 44 participants, 251 children were finally selected.

### 2.2. Questionnaires

#### 2.2.1. SDQ for Parents

The parent-rated SDQ consisted of 25 positively and negatively phrased items rated on a 3-point Likert scale (i.e., not true, somewhat true, or certainly true) [6]. The items were divided equally into five subscales (i.e., emotional symptoms, conduct problems, hyperactivity/inattention, peer relationship problems, and prosocial behavior). The total difficulty score (from 0 to 40) was generated from the sum of four subscale scores (i.e., emotional symptoms, conduct problems, hyperactivity/inattention, and peer relationship problems); the prosocial behavior subscale was not included in the total difficulty score. The specific subscale score (from 0 to 5) was calculated by adding the individual scores for each subscale. Except for the prosocial behavior subscale, higher scores indicated more problems. If a score on one of the constructs was missing, the total score was not calculated [6].

The cutoffs designed for the Japanese population were adapted from the study by Matsuishi et al. [11]. The scores were classified as follows: low need (0–12), some need (13–15), and high need (16–40). We considered “high need children” as children with behavioral problems and “low need children” as children without behavioral problems. Therefore, the 42 children who were considered as “some need children” were excluded.

#### 2.2.2. Educational Histories of Parents

To investigate the socioeconomic factors, the educational histories (in years) of the father and mother were investigated using a questionnaire.

#### 2.2.3. Picture Vocabulary Test–Revised

To assess vocabulary comprehension, the Picture Vocabulary Test–Revised (Nihon Bunka Kagakusha Co., Ltd., Tokyo, Japan) was used.

### 2.3. Physical Function

#### 2.3.1. One-Leg Standing Test

The one-leg standing test (OLST) was used to assess static balance. This was achieved by asking the participants to lift their feet for a maximum of 120 s. The time elapsed before participants were unable to maintain balance (for example, stepping or slipping) was recorded [12]. Measurements were taken twice, one for each leg. The maximum value was selected as the participant’s score.

#### 2.3.2. Two-Step Test

The two-step test was used to assess dynamic balance. This test was able to assess both dynamic balance and lower extremity muscle strength [13]. This was achieved by asking the participants to walk as far forward as possible for two consecutive steps and to stop with both feet together after the second step [14]. If they lost their balance or failed to stop after the second step, the test was repeated. The participants were also not allowed to jump. The test was conducted until two results were obtained. The maximum distance was selected and divided by the participant’s height.

#### 2.3.3. Five-Times-Sit-to-Stand Test

The five-times-sit-to-stand test measured the participants’ functional muscle strength. This was performed by asking the participants to sit on a chair with their arms crossed over their chests. The participants were then asked to stand and sit five times as quickly as possible without using the arm support [15,16]. The time taken to stand up for the fifth repetition was recorded.

### 2.4. Statistical Analysis

Power analysis for the Spearman’s rank correlation coefficient was performed using G* Power (Heinrich Heine University Düsseldorf, Düsseldorf, Germany) [17,18] to determine the optimal sample size with a statistical power of 0.95, an alpha of 0.005, and an effect size of 0.3 on two-tailed tests. The results of the power analysis showed that the required sample size was 205 participants.

The Shapiro–Wilk test was used to assess the normal distribution of each variable. The data were expressed as either mean ± standard deviation or median with the range. The independent *t*-test and Mann–Whitney U test were used to compare the two groups. The chi-square test was used to compare the differences between the boys and girls in each group. Statistical significance for group comparisons was set at *p* < 0.05.

Spearman’s rank correlation coefficients were used to determine the relationship between the physical functions that showed a significant difference between the two groups and the SDQ for all participants. In addition, the coefficients of correlation between the physical functions and the Picture Vocabulary Test–Revised were analyzed to check the effect of vocabulary comprehension on balance function. The statistical significance for the correlation analysis was set at *p* < 0.005. All analyses were performed using SPSS ver. 24 (IBM Inc., Armonk, NY, USA).

This study was conducted with the approval of the Ethical Review Committee of the Aichi Prefectural Mikawa Aoitori (REC number: 16000004; IRB approval number: 29002) and the Graduate School of Medicine, Nagoya University (REC number: 11001022; IRB approval number: 19-522). Written informed consent was obtained from the children and their guardians.

## 3. Results

A total of 209 children, both with (*n* = 38) and without (*n* = 171) behavioral problems, were eligible for this study. The demographic characteristics of the participants are presented in Table 1. In terms of the SDQ scores, emotional symptoms (*p* < 0.001), conduct problems (*p* < 0.001), hyperactivity/inattention (*p* < 0.001), peer relationship problems (*p* < 0.001), and total difficulties (*p* < 0.001) were significantly higher in children with behavioral problems than in those without. On the contrary, prosocial behavior (*p* = 0.046) was significantly lower in children with behavioral problems. There were significant sex differences (*p* < 0.001) between the two groups, with more male children than female children exhibiting behavioral problems. Meanwhile, significant differences were observed in neither the Picture Vocabulary Test–Revised scores nor the educational histories of the father and mother.

The physical characteristics of the participants are shown in Table 2. In terms of balance function, children with behavioral problems showed significantly smaller values on the OLST results (*p* < 0.001) and on the two-step test results (*p* = 0.008) than did those without. Meanwhile, no significant difference was observed in the five-times-sit-to-stand test results.

The correlations between the SDQ scores and the Picture Vocabulary Test–Revised scores and the physical function results for all participants are shown in Table 3. The OLST results were significantly correlated with emotional symptoms (r = −0.22, *p* < 0.001), hyperactivity/inattention (r = −0.29, *p* < 0.001), peer relationship problems (r = −0.22, *p* < 0.001), and total difficulties (r = −0.32, *p* < 0.001). Meanwhile, the two-step test results were not significantly correlated with the SDQ subscale scores, total difficulty, or the Picture Vocabulary Test–Revised scores.

## 4. Discussion

In this study, we demonstrated the characteristics of balance functions and their associations with the degree of behavioral problems in school-aged children. The OLST and two-step test results show significantly smaller values, suggesting a decrease in static and dynamic balance functions in children with behavioral problems compared to those without. The association between the OLST results and both the total difficulty and the SDQ subscale scores indicates that static balance might be related to behavioral problems, especially with regard to emotional problems, hyperactivity/inattention, and peer relationship problems. These results support our hypothesis that decreased balance functions are correlated with the degree of behavioral problems, but they are not unequivocal in our research. This highlights the importance of paying attention to both the balance and behavioral aspects of children with behavioral problems.

There is a significant correlation between the OLST results and the hyperactivity/inattention SDQ subscale scores. A previous study reported that children with mental restlessness experience greater swaying of the center of gravity than adults [19]. Another study suggests that children with behavioral problems pay less attention to tasks, which leads to poor motor function [20]. In light of these findings, it is expected that static balance (i.e., the ability to maintain a stationary posture) may be affected by the inherent hyperactive characteristic of children. Although the correlation is low, it may require attention.

Significant correlations were found between the OLST results and emotional symptoms. A previous study in adolescents and young adults supports the hypothesis that poor balance function is very stressful, such that it leads to problems of internalization [9,21]. In addition, children who are unable to cope with balance-threatening situations develop generalized anxiety and fear [22]. In the present study, it is suggested that the evaluation of emotional symptoms using the SDQ reflects anxiety and fear in prepubescent children. Furthermore, instability in the OLST evaluation induces anxiety and fear in children with high emotional symptom scores. We demonstrated an indirect relationship between balance function and emotional symptoms. Although the correlation between emotional symptoms and the static balance function in the SDQ is low, it is crucial to further investigate the relationship between balance function and other psychosocial factors.

Significant correlations were found between the OLST results and peer relationship problems. The relationship between motor function in daily life and peer relationships has been previously reported [9]. Furthermore, children with poor motor function are less likely to participate in organized and unorganized play [23]. In line with this, we assume that children with reduced static balance function are likely to develop peer relationship problems due to limited opportunities to play with peers. However, since the correlation between the peer relationship problems and the OLST results is low, the model of the relationship between peer relationship and static balance should be clarified in more detail by including multiple related factors.

It is possible that impaired balance function in children with behavioral problems is due not only to behavioral characteristics but also to somatosensory perception. It has been reported that balance is closely related to somatosensory perception in children [24]. Moreover, the OLST is a test that requires somatosensory perception [25]. The somatosensory input at the bottom of the foot could be one of the most essential components of one-leg balance control in children. Therefore, it is possible that the decline in static balance function in children with behavioral problems is also influenced by a decline in somatosensory perception.

In the comparison between the children with and without behavioral problems, there were significant differences in the two-step test results and sex. Since dynamic balance requires somatosensory perception and static balance, it is thought to be decreased in children with behavioral problems. However, compared with static balance, dynamic balance is more influenced by visual and vestibular functions, requiring predictive postural control. Due to these influences, it could be inferred that there is no correlation between the two-step test results and the SDQ scores. However, further investigation is still needed. Regarding the sex differences, a study among Japanese school-aged children showed that the total difficulty score in boys is higher than that in girls [11]. The results of the current study are consistent with this finding.

The finding that balance function is impaired in children with behavioral problems needs to be considered in clinical and educational situations. The focus tends to be on the emotional aspects of caring for children with behavioral problems. However, it is also important to reduce the risk of injuries and falls by paying attention to balance function when engaging in activities and physical tasks.

There was no significant correlation between the Picture Vocabulary Test–Revised scores, the OLST results, and the two-step test results in this study. A previous study of children aged 5 years reported that those with lower lexical development have more postural instability during open-eyed standing on a hard surface using stabilometry [26]. However, that study used different methods to assess static balance function and vocabulary skills from the present study, which may have impacted the differences in the results. The OLST assesses not only equilibrium, but also the muscle strength of the lower extremities. The Picture Vocabulary Test–Revised assesses vocabulary comprehension, while the Children’s Dictionary Test assesses vocabulary expression. In the future, we would like to investigate static balance function using stabilometry and clarify the relationship between static balance function and vocabulary ability.

Despite the promising results, this study has several limitations. First, due to the cross-sectional design, no clear causal relationship could be established. Second, since behavioral problems were categorized using a questionnaire, the subjective judgment of a parent or guardian could have influenced the results. Third, since the behavioral characteristics were only assessed using the SDQ, other potential causes of behavioral problems (e.g., autism spectrum disorder, attention deficit hyperactivity disorder, learning disorder, and developmental coordination disorder) could not be ruled out. Fourth, participants in this study may not have been representative of the general school-aged population because they live in particular area of Japan. Finally, since only the patient-answered SDQ was used, the behavior of the participants in social settings was not determined. As such, further investigations into the development of balance function and behavior from childhood to adolescence are needed.

## 5. Conclusions

In conclusion, we quantitatively evaluated balance functions and their associations with behavioral traits in school-aged children using the SDQ. Our findings suggest that static and dynamic balance functions are lower in children with behavioral problems than in those without behavioral problems. Furthermore, static balance function is associated with emotional, hyperactivity/inattention, and peer relationship problems. This suggests that the balance function of children with behavioral problems needs to be considered.

## Figures and Tables

**Table 1:** Demographic characteristics of the study population.

Variables	Group		*p*-Value
	Children with Behavioral Problems (*n* = 38)	Children without Behavioral Problems (*n* = 171)	
Age (years)	8.5 (6.0–10.0)	9.0 (6.0–10.0)	0.241
Sex (*n*, female/male)	12/26	109/62	<0.001
Height (cm)	127.0 ± 8.0	129.6 ± 9.1	0.104
Weight (kg)	24.0 (18.0–44.3)	25.8 (16.1–45.1)	0.184
Body Mass Index (kg/m^2^)	15.2 (12.8–26.6)	15.3 (12.3–23.6)	0.465
Picture Vocabulary Test–Revised (score)	10.0 (1.0–19.0)	11.0 (3.0–19.0)	0.373
Educational histories of father (years)	15.0 (9.0–20.0)	16.0 (9.0–21.0)	0.178
Educational histories of mother (years)	14.0 (9.0–21.0)	14.0 (9.0–21.0)	0.401
SDQ (score)			
Emotional symptoms	5.0 (0.0–9.0)	1.0 (0.0–7.0)	<0.001
Conduct problems	4.0 (1.0–8.0)	1.0 (0.0–4.0)	<0.001
Hyperactivity/inattention	8.0 (1.0–10.0)	3.0 (0.0–8.0)	<0.001
Peer relationship problems	3.0 (0.0–8.0)	1.0 (0.0–5.0)	<0.001
Prosocial behavior	6.0 (1.0–9.0)	7.0 (0.0–10.0)	0.046
Total difficulties	18.0 (16.0–34.0)	7.0 (1.0–12.0)	<0.001

The significance level was set at 0.05. Data are presented as mean ± standard deviation or median (min–max). Comparisons between groups were carried out using the independent *t*-test or the Mann–Whitney U Test. Sex differences were assessed by the chi-square test. SDQ: Strength and Difficulties Questionnaire.

**Table 2:** Differences in the physical function results between children with and without behavioral problems.

Variables	Group		*p*-Value
	Children with Behavioral Problems (*n* = 38)	Children without Behavioral Problems (*n* = 171)	
One-leg standing test (sec)	62.3 (15.6–120.0)	120.0 (13.1–120.0)	<0.001
Two-step test	1.5 (1.3–1.8)	1.6 (0.8–1.9)	0.008
Five-times-sit-to-stand test (sec)	5.7 (4.0–10.0)	5.5 (3.4–9.5)	0.692

The significance level was set at 0.05. Data are presented as mean ± standard deviation or median (min–max). Comparisons between groups were carried out using the independent t-test or the Mann–Whitney U test.

**Table 3:** Correlation between the SDQ scores and the Picture Vocabulary Test–Revised scores and the physical function results in all children.

Variables	OLST		Two-Step Test	
	Coefficient of Correlation	*p*-Value	Coefficient of Correlation	*p*-Value
SDQ				
Emotional symptoms	−0.22	<0.001	−0.16	0.021
Conduct problems	−0.19	0.006	−0.08	0.233
Hyperactivity/inattention	−0.29	<0.001	−0.16	0.021
Peer relationship problems	−0.22	<0.001	−0.10	0.167
Prosocial behavior	0.18	0.008	−0.03	0.717
Total difficulties	−0.32	<0.001	−0.18	0.009
Picture Vocabulary Test–Revised	0.02	0.762	0.07	0.288

The significance level was set at 0.005. This analysis was performed using the Spearman’s rank correlation coefficient. SDQ: Strength and Difficulties Questionnaire; OLST: one-leg standing test.

## Data Availability

All relevant data are presented within the manuscript. All data are available from the corresponding author on reasonable request.
